# Using Social Network Analysis to Assess Mentorship and Collaboration in a Public Health Network

**DOI:** 10.5888/pcd12.150103

**Published:** 2015-08-20

**Authors:** Miruna Petrescu-Prahova, Basia Belza, Katherine Leith, Peg Allen, Norma B. Coe, Lynda A. Anderson

**Affiliations:** Author Affiliations: Basia Belza, Norma B. Coe, University of Washington, Seattle, Washington; Katherine Leith, University of South Carolina, Columbia, South Carolina; Peg Allen, Washington University in St. Louis, St. Louis, Missouri; Lynda A. Anderson, Centers for Disease Control and Prevention and Emory University, Atlanta, Georgia.

## Abstract

**Introduction:**

Addressing chronic disease burden requires the creation of collaborative networks to promote systemic changes and engage stakeholders. Although many such networks exist, they are rarely assessed with tools that account for their complexity. This study examined the structure of mentorship and collaboration relationships among members of the Healthy Aging Research Network (HAN) using social network analysis (SNA).

**Methods:**

We invited 97 HAN members and partners to complete an online social network survey that included closed-ended questions about HAN-specific mentorship and collaboration during the previous 12 months. Collaboration was measured by examining the activity of the network on 6 types of products: published articles, in-progress manuscripts, grant applications, tools, research projects, and presentations. We computed network-level measures such as density, number of components, and centralization to assess the cohesiveness of the network.

**Results:**

Sixty-three respondents completed the survey (response rate, 65%). Responses, which included information about collaboration with nonrespondents, suggested that 74% of HAN members were connected through mentorship ties and that all 97 members were connected through at least one form of collaboration. Mentorship and collaboration ties were present both within and across boundaries of HAN member organizations.

**Conclusion:**

SNA of public health collaborative networks provides understanding about the structure of relationships that are formed as a result of participation in network activities. This approach may offer members and funders a way to assess the impact of such networks that goes beyond simply measuring products and participation at the individual level.

## Introduction

Chronic diseases are the main causes of poor health, disability, and death and account for most health care expenditures in the United States (1). The complexity of addressing chronic disease burden requires individuals and organizations to engage in collaborative networks that promote systemic changes and enhance community capacity and mobilization (2–4). Recognizing the importance of collaborative networks for chronic disease prevention and control, especially for older adults, the Centers for Disease Control and Prevention’s (CDC’s) Healthy Aging Program created the Healthy Aging Research Network (HAN), a partnership of leading academics, practitioners, and policy makers with expertise in aging and public health (5). Funded from 2001 through 2014, the network engaged community partners and funders to examine determinants of healthy aging, identify interventions that promote healthy aging, and disseminate information via community-based programs (6). The goals of the HAN were to 1) collaboratively develop a healthy aging research agenda, 2) facilitate partnerships to strengthen aging research, and 3) establish national linkages resulting in the potential to develop and promote interventions at individual, organizational, environmental, and policy levels (7).

In this study, we examined the ability of the HAN to create a cohesive community of diverse stakeholders by conducting a social network analysis (SNA) of the mentorship and collaboration relationships among its members. SNA is a systems science methodology (8) that focuses on relationships among individuals or organizations instead of solely on their attributes (9,10). As such, it can be used to examine the overall level of involvement of members in the network and particular patterns of involvement (3). Our results and approach may inform the assessment of other collaboration networks aimed at promoting population health at the local, state, and national levels.

## Methods

We used traditional SNA methods (9) to examine mentorship and collaboration in the HAN (Table 1) and collected cross-sectional data during January 2014. The University of Washington Institutional Review Board approved the study.

**Table 1:** Steps for Designing a Complete-Network^a^ Study or Evaluation

Social Network Analysis Step	Healthy Aging Research Network (HAN) Application
Define boundaries of the group	97 HAN members and partners identified as currently active in the network
Define relationship(s) of interest in conjunction with network stakeholders	Mentorship, collaboration on 6 types of products (ie, published papers, in-progress manuscripts, grant applications, community assessment tools or other data collection instruments, research projects, and presentations)
Collect data on the relationship(s) through surveys, from archival sources, or both	Online survey that included a roster of HAN members and partners
Create visualization(s) of the network	Individual level (Figure 1) and organizational level (Figure 2)
Analyze the network using social network analytical methods	Calculation of HAN network-level indices (9): Density: fraction of observed links to total number of possible links Isolates: actors not connected to the rest of the network Components: subgroups of actors connected among themselves but disconnected from the rest of the network Centralization: the extent to which the network is dominated by a central actor

a In a complete-network design, all members of the target group are surveyed to collect data about their relationships with other group members.

### Network membership and recruitment procedures

We used a “complete network” research design, which refers to an examination of the relationships among all actors in a given group (11). In January 2014 the HAN consisted of 7 member centers and several partner organizations. Member centers were Prevention Research Centers (PRCs) funded by CDC’s PRC Program (www.cdc.gov/prc), one of which served as a coordinating center. Partner organizations included affiliate (ie, not funded) academic centers, CDC, and other organizations focused on aging (eg, AARP, Administration for Community Living, National Council on Aging) (6). Member centers and partner organizations were represented by one or more individuals, whom we refer to as “members” and “partners.” The coordinating center project manager compiled a list of the 97 current members and partners, who were all invited to participate in the study. The invitation was sent by email and was followed by 2 email reminders at 10-day intervals.

### Relationship definition and measurement

In SNA, the kinds of relationships measured are defined by the researcher in conjunction with network stakeholders to align with the goals of the project and the characteristics of the group studied. Preliminary discussions with HAN members suggested that mentorship and collaboration were the 2 relationships that best defined the goals and unique character of the HAN. Consequently, our first objective was to capture the ongoing exchange of expert mentoring between members. Mentoring is a developmental relationship between 2 colleagues in which one person has more experience or authority than the other. Mentoring may include helping another person with improving work skills, understanding organizational history of the work context, providing information about advancing in the job or profession, and giving personal or emotional support (12). This relationship can occur formally, such as between advisor and advisee, or informally. Our second objective was to measure collaboration on 6 products that helped quantify the activities of the network: published papers, in-progress manuscripts, grant applications, tools, research projects, and presentations. Collaboration is formally defined as an interaction taking place within a social context between 2 or more partners that facilitates the sharing of knowledge and completion of tasks with respect to a mutually shared goal (13).

Data collection instruments in SNA can include open-ended or closed-ended items (11). The closed-ended format, in which a respondent completes the same set of items about his or her relationships with each network member from a provided roster, is best used when the boundaries of the network (and therefore its member list) are well defined, as was the case for our study. Furthermore, this format raises fewer concerns about respondent recall and accuracy than the open-ended (or “name generator”) format, in which respondents are asked to list people who they, for example, “collaborate with” or “are mentoring” (11).

We developed an online SNA data collection instrument with 8 closed-ended items (Appendix). We piloted the instrument with 4 HAN members and revised some items on the basis of their comments. We included definitions for mentorship and collaboration in the survey to facilitate a common understanding of the concepts in the context of the study and ensure validity. We provided respondents with a roster of the 97 HAN members and partners and asked them to report on mentorship and collaboration with each of them in the previous 12 months. This time limit was used to reduce recall bias (11). The questionnaire also included items about length of involvement in the HAN; the coordinating center project manager provided data on organizational affiliation. In the remainder of this article, we use the term “mentorship network” to refer to the observed mentorship relationships among HAN members and partners. “Collaboration networks” is used to refer to the observed collaboration relationships among HAN members and partners on different types of products.

### Data analysis

Network visualization (14) is a form of exploratory analysis, which is similar to the scatterplots and histograms employed in traditional statistical analyses that describe the distribution of a sample. It allows network mapping through a visual representation of the actors as points and ties as lines among points; such maps are called sociograms. Sociograms depict existing ties and can include additional actor attributes represented through the color, shape, and size of the points and tie attributes through the direction, color, and thickness of the lines. The position of the points in such plots is determined through an algorithm that places points that have many ties to other points toward the center of the plot and less well-connected points toward the periphery, while trying to maintain equal line length. For directional relationships that presuppose a “sender” and a “receiver,” the lines have arrows to indicate the direction.

To describe the structural patterns of the HAN mentorship and collaboration networks, we calculated the following network-level indices: density, proportion of isolates, number of components of size greater than 1, and centralization (9). Network-level indices are useful because they allow for comparisons among different networks (11). Density is calculated as the fraction of observed ties to total number of possible ties and is valued between 0 and 1. Density is the most widely used measure of network cohesion; a value closer to 0 indicates a sparse network, and a value closer to 1 indicates a tightly knit network in which almost all pairs of actors are connected. Isolates are actors who are not connected to the rest of the network; components are subgroups of actors who are connected among themselves but are disconnected from the rest of the network (isolates are a component of size 1). These 2 measures reflect the amount of fragmentation in the network. Centralization measures the extent to which the network is dominated by a central (or prominent) actor and also is valued between 0 and 1. The centrality of an actor can be measured in various ways, such as the number of connections an actor has to others (degree centrality) or the proportion of shortest paths between pairs of actors that include a certain actor (betweenness centrality). We used these 2 conceptualizations of centrality to calculate degree and betweenness centralization. A value of centralization closer to 0 indicates a decentralized network, in which actors have similar levels of centrality or importance, and a centralization value closer to 1 indicates a highly centralized network where one or more actors dominate in terms of having more ties to network members or higher betweenness compared with others. For mentorship, which, in contrast to collaboration, is a directional relationship, we also calculated reciprocity, the proportion of ties in the network that are reciprocated or mutual.

We created sociograms of the HAN mentorship and collaboration networks using the specialized SNA software UCINET (15) and computed the above network-level measures using the SNA package of the R statistical software (14). We used descriptive statistics to report individual-level characteristics of respondents.

We considered a tie to be present if one of the actors connected by the tie reported it as present. Therefore, we were able to capture some data about the 34 HAN members who did not respond or provided incomplete data on mentorship and collaboration relationships by using other respondents’ answers. For example, a respondent could indicate collaborating with or receiving mentorship from a HAN member or partner, even if the latter had not actually completed the survey. However, we could not capture information about relationships between nonrespondents.

## Results

Sixty-eight of the 97 HAN members and partners responded to the survey. Two respondents accessed the survey link but declined to participate, and another 3 did not identify themselves, bringing the number of complete responses to 63. This represents a response rate of 65%, which is high for Web-based surveys (16,17). Of the 63 respondents, 30 were from member centers (48%); all member centers were represented in the sample. Thirty-three respondents were partners (52%) — 4 from affiliate academic centers (6%), 8 from CDC (13%), and 21 from other aging organizations (33%). The average length of HAN involvement among the respondents was 6.6 years.

**Figure 1:**
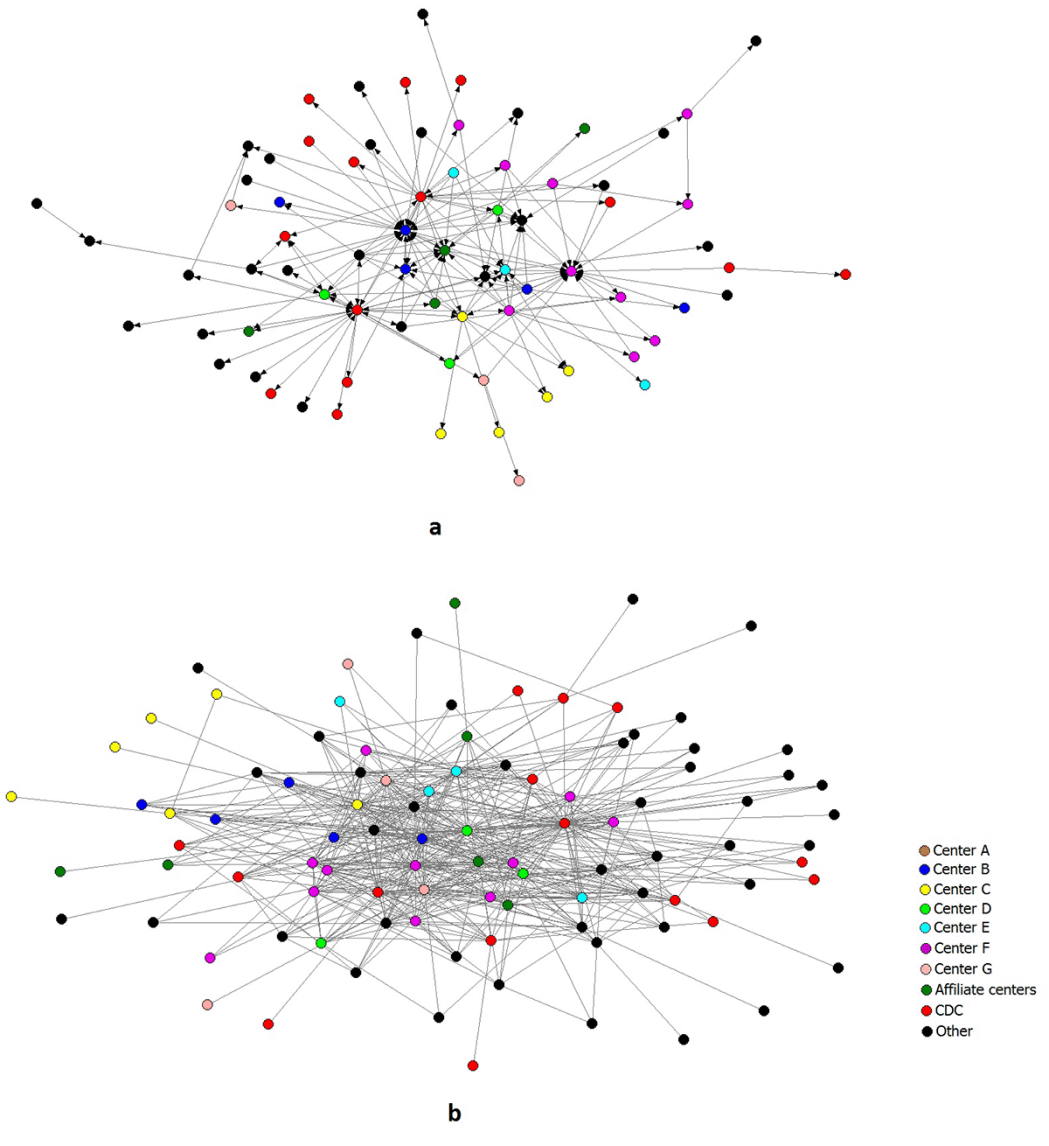
Sociograms of the individual-level mentorship and collaboration networks of the Healthy Aging Research Network members and partners, United States, January 2014.

Figure 1 shows sociograms of the HAN mentorship (1a) and collaboration (1b) networks at the individual level. The 97 HAN members and partners are represented through circles whose colors depend on the organization to which they belong. We labeled the 7 member centers with letters A through G and divided the rest of the actors into 3 categories: affiliate centers, CDC, and other.

The lines in Figure 1a represent mentorship offered or received by the actors. Although three-fourths of the 97 HAN members and partners were connected by mentorship ties, some of them were more involved in mentorship, sending or receiving a larger number of ties than others. In addition, 13.5% of the reported mentorship ties were reciprocated, which indicates that actors were both mentoring and being mentored by the same person.

The lines in Figure 1b represent collaboration on any kind of product. All HAN members and partners were connected by at least one form of collaboration on products. At the same time, the network displays what is commonly referred to as a “core-periphery” structure (11) — a dense, cohesive core and a sparse, unconnected periphery.

The collaboration network is almost twice as dense as the mentorship network (0.11 vs 0.06), showing that HAN members were more likely to be engaged in collaboration than mentorship. In terms of different types of collaboration, the most cohesive collaboration network (as measured by density) was on research projects, followed by presentations, and in-progress manuscripts (Table 2). Some collaboration networks, such as for tools and presentations, were more centralized, whereas others, such as for manuscripts and grant applications, were less centralized.

**Table 2:** Network-Level Indices for Mentorship and Collaboration Among Healthy Aging Research Network Members and Partners, United States, January 2014

Relationship	Density^a^	Proportion of Isolates^b^	Number of Components of Size at Least 2^c^	Degree Centralization^d^	Betweenness Centralization^e^
Mentorship	0.06	0.24	1	0.17^f^	0.07
Collaboration (any form)	0.11	0	1	0.52	0.28
Published papers	0.03	0.42	1	0.30	0.13
In-progress manuscripts	0.05	0.34	1	0.28	0.11
Grant applications	0.03	0.35	3	0.17	0.10
Tools	0.04	0.26	1	0.50	0.37
Research projects	0.07	0.16	1	0.33	0.16
Presentations	0.05	0.18	1	0.42	0.29

a Density is calculated as the fraction of observed ties to total number of possible ties and is valued between 0 and 1.

b Isolates are actors who are not connected to the rest of the network.

c Components are subgroups of actors who are connected among themselves but are disconnected from the rest of the network.

d Degree centralization measures the extent to which the network includes actors who have many ties to other actors and takes values between 0 (all actors have same number of ties) and 1 (only ties are between 1 actor and all other actors — star configuration) (9).

e Betweenness centralization measures the extent to which the network includes actors who are on the shortest paths between other pairs of actors. It takes values between 0 (all actors are connected to all other actors directly) and 1 (only ties are between one actor and all other actors — star configuration) (9).

f Calculated based on total number of ties sent and received by actors, because mentorship is a directed relationship.

**Figure 2:**
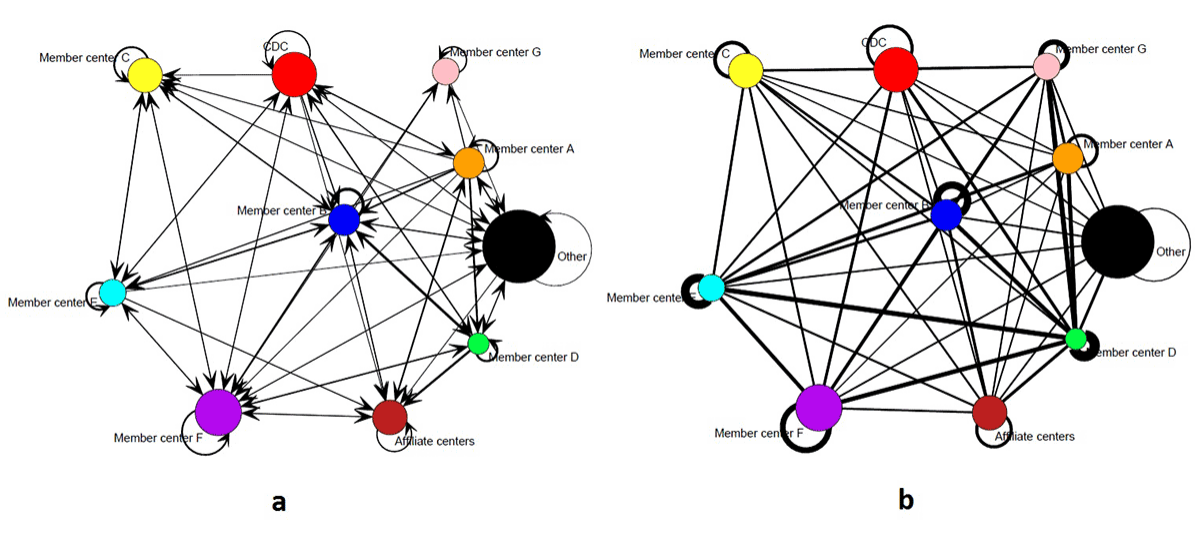
Sociograms of the mentorship and collaboration networks of the Healthy Aging Research Network members and partners aggregated at the organizational level, January 2014.

We also examined patterns of mentorship and product collaboration at the organizational level, between member centers and partner organizations. Figure 2 shows sociograms of the HAN mentorship (2a) and collaboration (2b) networks aggregated at the organizational level. These sociograms have 10 nodes each, corresponding to 7 member centers (labeled A through G), CDC, affiliate academic centers (1 node), and other aging organizations (1 node). The size of the nodes is proportional to the number of actors in each category, and the thickness of the lines is proportional to the number of ties that exist between organizations. These sociograms also include self-ties (loops), because mentorship and collaboration can exist between members of the same organization. In both aggregate networks all nodes are connected to many other nodes, and all possible self-ties are present. Moreover, in the case of product collaboration, all possible ties are present, which indicates that mentorship and product collaboration ties developed both within and between HAN member and partner organizations.

## Discussion

We conducted an SNA of mentorship and collaboration relationships among members and partners of the HAN. Our results show high connectivity among HAN members and partners across organizational boundaries, although there was substantial variation in the level of individual involvement. These results indicate that the HAN has been successful in creating mentorship and collaboration linkages among a diverse group of healthy-aging stakeholders.

The benefits of mentoring have long been recognized. Mentoring is key to the development of leaders, because it contributes to role and career development of the mentee and provides both mentee and mentor with opportunities for interpersonal and professional growth and career satisfaction (18). Most HAN members and partners were mentors to, or were mentored by, another member or partner. Moreover, 13.5% of these relationships were reciprocated. Although this value seems low compared with other types of relationships that are easily reciprocated, such as friendship or transmission of information (9), our definition for mentorship was based on a status or expertise differential. In such cases, the expected structure of the network is hierarchical and contains no reciprocated ties or cycles (19). The presence of reciprocated ties in this network suggests that the HAN was successful in bringing together researchers and practitioners with different areas of expertise and at different stages of their careers who were actively learning from one another.

Respondents reported being engaged in at least one form of collaboration as a result of participation in the HAN. This suggests that the HAN was an active, cohesive community that was able to bring together researchers and practitioners to collaborate on programs and policies. This result may be a consequence of the fact that the HAN met the 5 conditions of collective impact that successful collaborative initiatives seem to possess: a common agenda, shared measurement, mutually reinforcing activities, ongoing communication, and backbone support (6,20). For HAN members and partners, the presence of a backbone support — the HAN coordinating center, which led, organized, managed, and synchronized the activities of the HAN — was valuable because it facilitated greater cross-sector alignment and learning among many organizations, inclusion of corporate and government sectors as essential partners, active coordination of HAN actions, and sharing of lessons learned (5).

Our study has limitations. Our response rate was lower than desirable for a complete network study, which preferably should be around 80% (21). As a consequence, our results likely underestimate the total amount of mentorship and collaboration, because we did not have complete information about ties among HAN members who did not respond. Conversely, they may overestimate the overall cohesiveness of the networks because of selection bias; survey respondents may have been more committed to the network and therefore more connected. Moreover, this study examined only a cross-sectional view of the activities of the HAN during the previous 12 months. Therefore, this study did not capture the relationships that had existed in the first 13 years of the HAN. Additionally, our study was conducted in the 14th year of HAN’s existence, capturing the network’s peak activity (5). Longitudinal data collection at various points during the life of the network would have allowed a more accurate examination of the evolution of mentorship and collaboration over time.

Chronic disease prevention is among the most complex public health issues our nation faces (1). As a result, decision makers and funders should bring together stakeholders with different expertise and areas of practice to address this problem with scarce public resources. Collaborative networks are valuable because they may lead to better outcomes by leveraging resources and finding solutions that are not achievable by a single organization (22). Examples of such networks abound; collaborative public health networks have been active in the areas of building community capacity for provision of chronic disease services (2), tobacco control (3,4,23), cancer screening (24), and obesity prevention (25). Furthermore, the CDC PRC program currently has 7 thematic research networks that focus on the healthy brain, cancer prevention and control, nutrition and obesity policy, workplace health promotion, epilepsy, physical activity, and global health.

At the same time, decision makers and funders need to be able to assess these networks with tools that match their complexity. Researchers have long argued for the increased use of systems science methodologies, among them SNA, as a way to examine emergent properties of systems such as collaborative networks — phenomena that are observed at the system level but cannot be causally linked to a specific individual component (26). However, these approaches remain underused (27). Future research may benefit from quantifying the benefits of network participation, assessing the relationship between level of involvement and benefits, and examining the relationship between network structure and outcomes (28). Furthermore, research could benefit from including measures about knowledge creation and knowledge transfer and then weighing them against the perceived costs of participation. Such approaches would offer decision makers and funders more adequate tools for evaluating the impact of public health collaborative networks.

## Appendix Healthy Aging Research Network Social Network Analysis Survey

This file is available for download as a Microsoft Word document.
