# Supplementary material for: Using Social Network Analysis to Assess Mentorship and Collaboration in a Public Health Network
Source: Prev Chronic Dis. 2015 Aug 20;12:E130. doi: 10.5888/pcd12.150103 (PMC4565512; doi:10.5888/pcd12.150103)
Supplement: Supplementary file 1 [file 15_0103_Appendix.docx]

Appendix

C. Mentorship and Collaboration

Mentoring is a developmental relationship between two colleagues in which one person has more experience or authority than the other. Mentoring may include helping another person with improving work skills, understanding organizational history of the work context, providing information about “getting ahead” in the job or profession, and giving personal or emotional support (Feeney and Bozeman, 2008). This relationship can occur formally, such as between advisor and advisee, or informally.

Collaboration is an interaction taking place within a social context between two or more partners that facilitates the sharing of knowledge and completion of tasks with respect to a mutually shared goal (Sonnenwald, 2007). Within the context of HAN, collaboration can be quantified using a number of different products such as manuscripts, publications, or grant applications.

In the following section, select the HAN members and partners you are mentoring or are being mentored by, as well as those with whom you have collaborated on the listed products within the past 12 months. Include products that are not related to HAN activities but are the result of a relationship or collaboration formed via HAN introduction.

Our primary aim is to identify relationships and productivity that have developed directly as a result of HAN participation.

Please list your mentorship and/or collaborations with the following HAN members and partners:

| Name | Mentor to | Mentored by | Published papers | Manuscripts (in progress) | Grant applications | Research projects | Presentations | Tools |
| --- | --- | --- | --- | --- | --- | --- | --- | --- |
| Person 1 |  |  |  |  |  |  |  |  |
| Person 2 |  |  |  |  |  |  |  |  |
| Person 3 |  |  |  |  |  |  |  |  |
| … |  |  |  |  |  |  |  |  |
